# Serum Procalcitonin as an Indicator of Disease Severity and Clinical Deterioration in Hospitalized Patients With Acute Cholangitis

**DOI:** 10.7759/cureus.89748

**Published:** 2025-08-10

**Authors:** Landon A Kozai, Yoshito Nishimura, Torrey Czech, Arvin Jeremy N Tan, Kevin Benavente, Krixie Silangruz, Larissa Fujii-Lau

**Affiliations:** 1 Internal Medicine, John A. Burns School of Medicine, University of Hawaiʻi, Honolulu, USA; 2 Oncology, Mayo Clinic, Rochester, USA; 3 Gastroenterology and Hepatology, California Pacific Medical Center, San Francisco, USA; 4 Gastroenterology and Hepatology, The Queen's Medical Center, Honolulu, USA

**Keywords:** ascending cholangitis, cholangitis, endoscopic retrograde cholangiopancreatography (ercp), hepatobiliary, procalcitonin

## Abstract

Background: Acute cholangitis (AC) results from bile stasis and subsequent infection in the biliary tract, requiring urgent evaluation to determine the timing of biliary decompression. Procalcitonin (PCT) and lactic acid (LAC) may be reliable indicators of disease severity to suggest early biliary decompression. We seek to assess the utility of PCT and LAC to predict severe AC and adverse in-hospital outcomes such as ICU transfer, vasopressor use, or mortality.

Methods: This is a retrospective observational study of patients admitted with AC to a tertiary hospital in the United States between January 1, 2016, and December 31, 2020. Patients were identified using International Statistical Classification of Diseases and Related Health Problems, Tenth Revision (ICD-10) codes for AC. Patients who had both PCT and LAC measured upon presentation were included. Logistic regression analysis was performed to determine the diagnostic performance of PCT and LAC to predict the outcomes.

Results: A total of 193 patients, accounting for 229 hospital encounters, were identified. Choledocholithiasis was the most common cause of biliary obstruction (31.9%), followed by mass or malignancy (21.0%). Endoscopic retrograde cholangiopancreatography* *(ERCP) was performed in 40.2% of patients. Percutaneous transhepatic biliary drainage and conservative management occurred in 21.8% and 34.5% of patients, respectively. PCT was non-superior to LAC and had low predictive values for the prediction of in-hospital mortality, ICU admission, or need for early decompression. In a subgroup analysis of malignancy-related cholangitis, PCT was superior to LAC in predicting in-hospital mortality (area under the receiver operating characteristic curve (AUROC) 0.932), ICU admission (AUROC 0.730), and need for early decompression (AUROC 0.767).

Conclusions: The findings of this study do not support the routine use of PCT in the evaluation of non-malignancy-related AC. PCT may be useful as a predictor of poor in-hospital outcomes in patients with malignancy-related AC. Further research is required to clarify its role in the evaluation of patients with AC.

## Introduction

Acute cholangitis (AC) is a clinical syndrome characterized by fever, jaundice, and abdominal pain that develops because of stasis and subsequent infection in the biliary tract. The Tokyo Guidelines 2018 for Acute Cholangitis (TG18) is a practical guideline that clearly defines the diagnostic criteria, severity assessment, and management for cholangitis [1]. The guidelines also suggest that the severity of AC can be classified into three grades, mild (grade I), moderate (grade II), and severe (grade III), on the basis of two clinical factors: the presence of organ dysfunction and the response to the initial medical treatment. The TG18 severity grading criteria for AC are important for predicting prognosis and determining a treatment strategy, especially in identifying patients who require early biliary drainage. 

Procalcitonin (PCT) is an inducible serum prohormone released from tissues by proinflammatory molecules and is highly predictive of bacterial sepsis above a threshold of 0.5 ng/mL [2-4]. Lower levels of PCT (<0.25 ng/mL or <0.1 ng/mL), depending on the site of infection, have a high negative predictive value for ruling out infection. In addition to its role in navigating the differential diagnosis of systemic inflammation, data show PCT is also useful to guide antibiotic therapy and antimicrobial stewardship in various infections [2-4]. Lactic acid (LAC) is a byproduct of anaerobic metabolism, often used as a prognostic tool in the diagnosis and management of sepsis [5]. Both PCT and LAC have been shown to be reliable indicators of disease severity in patients with infection [6,7].

Research indicates that measurement of serum PCT may provide crucial prognostic information in patients with AC. PCT was found to be elevated in AC and correlated with disease severity when assessed at varying biomarker cutoffs [8-10]. In particular, PCT predicted severe AC and eventual clinical deterioration to septic shock [10,11]. Another small study found that PCT was a prognostic factor for the development of AC in patients with acute pancreatitis [9].

The role of PCT in the diagnosis and management of AC remains unclear. The TG18 guidelines suggest that PCT is a useful parameter for severity assessment of AC, although evidentiary support is only drawn from three case series, and therefore, the guidelines recommend further investigation of PCT [12]. Two of three cases found that PCT may be able to discriminate between AC severity grades, while a third concluded that a cutoff value of 2.2 ng/mL predicted severe AC better than white blood cell count and C-reactive protein [8,10,13]. These were all small, single-center studies focusing solely on AC secondary to choledocholithiasis and excluded patients with a percutaneous biliary catheter in situ or prior treatment. In-hospital outcomes such as mortality or ICU admission were not assessed, and as such, the question of whether the use of PCT to guide clinical decision-making leads to improved in-hospital outcomes remains unanswered. There is also a lack of data to guide clinical decision-making in patients who develop AC from other causes, such as those with AC due to malignant biliary strictures. In our study, we sought to expand upon this body of evidence by broadening the inclusion criteria to AC with any causes.

A recent review concluded that there were also inconsistencies in the grading of AC severity across these studies, owing to the use of different versions of the Tokyo Guidelines criteria, and that the definition of “urgent” biliary decompression was also heterogeneous [14]. It also noted that these studies lacked subgroup analyses to define the use of PCT in different etiologies of AC. While choledocholithiasis accounts for a majority of AC cases, 10-30% of AC cases are attributable to malignant biliary stricture [15]. Therefore, we set out to further evaluate the use of PCT in diverse populations and assess the ability of PCT to predict severe AC and adverse in-hospital outcomes such as ICU transfer, vasopressor use, or mortality. We hypothesize that PCT is superior to LAC in predicting these outcomes.

## Materials and methods

This is a retrospective observational study of a review of electronic health records at the Queen’s Medical Center in Honolulu, Hawaii, United States. The study period was between January 1, 2016, and December 31, 2020. The study was approved by the institutional review board of The Queen’s Medical Center, Honolulu, Hawaii (reference number: RA-2022-036, dated October 20, 2022). All research methods were performed in accordance with relevant guidelines and regulations

Study subjects

A consecutive cohort of patients over the age of 18 who were admitted to a single tertiary referral hospital with International Statistical Classification of Diseases and Related Health Problems, Tenth Revision (ICD-10) codes for the diagnosis of acute cholangitis were considered for inclusion in the study. Patients were excluded from the study if serum PCT and serum LAC were not measured at presentation, and if they were concurrently diagnosed with other potential sources of infection except for acute cholecystitis, because of its close association with AC.

Data collection

Demographic data, including age, sex, race and ethnicity, select comorbidities, vital signs on initial contact with the emergency room, and routine laboratory data from the initial 24 hours of presentation, including basic metabolic panel, complete blood count, and hepatic function profile, LAC, and PCT, were collected through retrospective chart review. Imaging results, ICU admission, vasopressor requirement, length of hospital stay, method of biliary decompression, etiology of AC, and time to biliary decompression were collected in a similar fashion.

Definitions

The severity of AC was determined based on the TG18 criteria using the available data. Patients underwent endoscopic retrograde cholangiopancreatography (ERCP), percutaneous transhepatic biliary decompression (PTBD), surgery, or no biliary decompression procedure at the discretion of the treating physician, and this was verified through manual chart review.

Statistical analysis

Logistic regression analysis was performed to determine the diagnostic performance of PCT and LAC to predict the outcomes. The statistical analyses were conducted with JMP Version 15.1 (SAS Institute, Cary, North Carolina, United States).

## Results

Baseline characteristics and demographics

A total of 193 patients who accounted for 229 hospital encounters were identified. The baseline characteristics of the 193 patients are presented in Table 1. The median age was 71 years, and the population was 62.0% male. The cohort was predominantly of Asian race (59.6%), followed by Native Hawaiian/Pacific Islander (17.1%) and Caucasian (10.9%) participants. The number of patients with a history of recurrent cholangitis secondary to hepatobiliary malignancy was 33 (17.1%). There were 56 (29.0%) and 27 (14.0%) patients who presented with a biliary stent or external biliary drain in place, respectively. Only 14 (7.3%) patients had cirrhosis, while 49 (25.4%) patients were concurrently diagnosed with acute kidney injury (AKI). The median PCT level was 1.8 ng/mL (0.49-6.8 ng/mL), and the median LAC was 2.0 mmol/L (1.5-2.8 mmol/L).

**Table 1:** Baseline characteristics and laboratory values on admission of the included patients (N=193) HPB, hepatopancreatobiliary

Parameters	Values
Age (years), median (IQR)	71.0 (63.0–79.0)
Sex, n (%)	
Male	120 (62.0)
Female	73 (36.0)
Race, n (%)	
Japanese	51 (26.4)
Filipino	39 (20.2)
Native Hawaiian/Pacific Islander	33 (17.1)
Chinese	25 (13.0)
Caucasian	21 (10.9)
Others	24 (12.4)
Comorbidity and relevant history, n (%)	
Prior biliary stent	56 (29.0)
Acute kidney injury	49 (25.4)
Recurrent cholangitis related to HPB malignancy	33 (17.1)
Prior external biliary drain	27 (14.0)
Chronic kidney disease	29 (15.0)
Cirrhosis	14 (7.3)
Laboratory values, mean (IQR)	
Procalcitonin (ng/mL)	1.8 (0.49–6.8)
Lactic acid (mmol/L)	2.0 (1.5–2.8)

Clinical characteristics and etiology of obstruction

Clinical characteristics of the 229 hospital encounters are presented in Table 2. The number of patients with stage 1, stage 2, and stage 3 cholangitis according to the TG18 criteria was 55 (24.0%), 141 (61.6%), and 33 (14.4%), respectively. Choledocholithiasis was the most common cause of biliary obstruction (31.9%), followed by mass or malignancy (21.0%), indwelling stent dysfunction (16.2%), and percutaneous drain dysfunction (6.6%). The cause of obstruction was unclear in 14.4%.

**Table 2:** Clinical characteristics and outcomes of 229 hospital encounters ERCP, endoscopic retrograde cholangiopancreatography; ICU, intensive care unit; PTBD, percutaneous biliary drainage; TG, Tokyo Guidelines *Of the 92 participants who underwent ERCP, 56 had sphincterotomy

Parameters	Frequency (Percentage)
TG18 Cholangitis Grade	
Stage 1	55 (24.0)
Stage 2	141 (61.6)
Stage 3	33 (14.4)
Interventions	
ERCP	92/229 (40.2)
ERCP (With sphincterotomy)	56/92 (60.9)*
PTBD	50 (21.8)
Surgery	8 (3.5)
No intervention	79 (34.5)
Time until decompressive intervention	
≤ 12 hours from presentation	25 (10.9)
12–24 hours from presentation	48 (21.0)
24–48 hours from presentation	37 (16.2)
> 48 hours from presentation	40 (17.5)
Cause of obstructions	
Stone	73 (31.9)
Mass/malignancy	48 (21.0)
Indwelling stent dysfunction	37 (16.2)
Percutaneous drain dysfunction	15 (6.6)
Sludge	10 (4.4)
Passed stone	7 (3.1)
Unclear	33 (14.4)
Death	16 (7.0)
ICU admission	48 (21.0)
Vasopressor	8 (3.5)
Endotracheal intubation in the first 24 hours	7 (3.1)

Biliary drainage method and timing

A summary of the timing of biliary decompression and chosen drainage method is also provided in Table 2. The majority of patients underwent ERCP (40.2%), and 60.9% of those undergoing ERCP also had sphincterotomy performed. Percutaneous transhepatic biliary drainage was performed on 21.8% of patients, and surgery in 3.5%. A large portion of patients had no intervention to relieve biliary obstruction performed as they improved without intervention (34.5%). In those undergoing intervention, biliary decompression occurred most often within 12-24 hours from presentation (21.0%). Emergent decompression occurring <12 hours from presentation only occurred in 10.9% of cases.

Serum procalcitonin as an indicator of worse in-hospital outcomes

The correlation between PCT and key clinical outcomes is summarized in Table 3. PCT had low predictive values for the prediction of in-hospital mortality at a cut-off value of 0.60 (area under the receiver operating characteristic curve (AUROC) 0.479), ICU admission (AUROC 0.601), need for early decompression ≤ 24 hours (AUROC 0.621), TG18 grade 3 disease (AUROC 0.616), or AKI (AUROC 0.682). PCT was non-superior to LAC in predicting these outcomes in patients without malignancy-related disease. In a subgroup analysis of malignancy-related AC, PCT was superior to LAC in predicting in-hospital mortality (AUROC 0.923) and ICU admission (AUROC 0.867). Furthermore, in patients with malignancy-related AC, PCT at a cut-off of 0.47 ng/mL yielded a sensitivity of 100% and specificity of 80.0% for the prediction of in-hospital mortality.

**Table 3:** Procalcitonin and lactic acid: univariate logistic regression analysis AUROC, area under the receiver operating curve; TG, Tokyo Guideline; ICU, intensive care unit; AKI, acute kidney injury

Parameters	Malignancy-related (AUROC)	Cut-off	Sensitivity	Specificity	Non-malignancy-related (AUROC)	Cut-off	Sensitivity	Specificity
Severe disease (TG18 Stage 3)								
Procalcitonin	0.625	1.8	100%	47.1%	0.616	0.47	62.5%	62.6%
Lactic acid	0.610	1.5	100%	38.2%	0.523	2.0	62.5%	54.2%
Mortality								
Procalcitonin	0.923	0.47	100%	80%	0.479	0.060	25%	99.3%
Lactic acid	0.505	2.4	100%	22.9%	0.560	1.3	100%	16.3%
ICU admission								
Procalcitonin	0.867	2.9	100%	78.8%	0.601	9.1	37.8%	83.9%
Lactic acid	0.624	0.624	80%	54.5%	0.716	3.3	54.1%	88.1%
AKI								
Procalcitonin	0.759	2,9	60%	86.9%	0.682	2.5	67.7%	64.6%
Lactic acid	0.425	7.4	6.7%	100%	0.724	2.6	70.6%	74.4%
Need for early Decompression (≤ 24 hours)								
Procalcitonin	0.752	0.98	100%	45.5%	0.621	1.4	73.7%	52.1%
Lactic acid	0.579	1.4	100%	24.2%	0.586	3.3	36.8%	82.9%

## Discussion

The TG18 guidelines propose a treatment pathway based on the initial disease severity assessment. The guidelines suggest that mild AC may be managed conservatively with fluids and antibiotics, while moderate to severe AC should be addressed with early biliary drainage. Mild AC, too, should be considered for biliary drainage if the patient fails to respond to initial conservative therapy. The availability of a biomarker to aid in severity assessment may potentially improve outcomes related to AC through early initiation of appropriate treatment pathways. Although prior research has shown promise in supporting the use of PCT for the triage of patients with AC [8,10,12,13], our data suggest it is a poor indicator of disease severity and in-hospital outcomes in patients without malignancy-related AC.

PCT may be a useful parameter in the evaluation of patients with malignancy-related AC due to its superiority over LAC for predicting in-hospital mortality. PCT was also superior to LAC in predicting the need for ICU admission. This suggests that PCT may be useful in the assessment of patients with malignant biliary strictures who have AC that are at higher risk for deterioration to septic shock and death. Recent data demonstrated that patients with moderate-to-severe AC due to primary distal malignant strictures were shown to have reduced mortality when treated with urgent ERCP (<24 hours) [16]. The incorporation of PCT into the evaluation of these patients may be helpful for determining the need for urgent ERCP.

The use of PCT in patients with cancer is an area of active study, and PCT in this case may play an important role in differentiating cancer-related inflammation from infection. Whereas C-reactive protein, white blood cell count, and sedimentation rate may be nonspecifically elevated in the setting of malignancy, a PCT level >0.50 ng/mL in the appropriate clinical context is sensitive and specific for bacterial infection, and elevations beyond 2 ng/mL are highly specific for severe sepsis [17]. Although PCT is viewed as a promising biomarker in the diagnosis and management of sepsis, it remains an area of controversy. Inconsistencies arise from meta-analyses concerning the diagnostic utility of PCT in sepsis, with conflicting evidence emerging. Furthermore, the appropriate population for PCT utilization remains uncertain; while its effectiveness has been demonstrated in critically ill patients, its applicability to the typical hospitalized patient remains unsubstantiated [3,18]. Moreover, PCT-guided clinical decision making did not reduce mortality, mechanical ventilation, clinical severity, reinfection, or duration of antimicrobial therapy in patients with septic conditions [19]. In general, a universal cut-off for interpretation of PCT level does not exist; its clinical application is complex and may differ with respect to the patient population, underlying comorbidity, and specific clinical context.

Still, it is suggested that tumor-induced inflammation does not alter PCT levels, and thus, PCT elevation could be an indicator favoring sepsis in patients with malignancy and signs of systemic inflammation [20-22]. However, some authors conclude that the use of PCT in patients with cancer is limited and requires further investigation [22]. In a study of 447 patients with non-hepatobiliary solid cancer and no ongoing infection, zero patients had measured PCT levels >0.50 ng/mL, which exceeds the cutoff identified in our study [21]. Also, the specified cut-off of 0.47 ng/mL is significantly below the median PCT level of 1.8 ng/mL, and in the context of this study population with an overall low incidence of mortality and severe cases, these results may not truly represent its diagnostic accuracy. Thus, although PCT could play an important role in clarifying the differential diagnosis and guiding treatment in patients with obstructive hepatobiliary cancer presenting with signs of systemic inflammation, further research is warranted, and clinicians should be aware of its limitations.

The study has several limitations. First, it is a single-center retrospective chart review predominantly including Asian and Native Hawaiian/Pacific Islander patients. Caucasian patients were a minority group, and the results may not be generalizable for this reason. Second, the sample was composed of a significant portion of patients who had undergone prior biliary drainage procedures and had either a biliary stent or a percutaneous biliary drain in situ. Cancer was the universal reason for having a drainage device in these patients. Interestingly, PCT was not predictive of in-hospital outcomes or AC severity in these patients. This may be related to earlier diagnosis and treatment in these patients compared to those with an index hospitalization due to mass or malignancy, due to more frequent monitoring, follow-up, and familiarity with early signs or symptoms. Patients with an undiagnosed mass or malignancy may present later in the clinical course of AC due to more indolent symptoms. Third, the sample size was relatively small, and the number of treatment-naive patients presenting with AC secondary to choledocholithiasis may have been insufficiently large for adequately powered statistical analysis. This could explain differences in results compared to other studies with strict inclusion criteria. Fourth, the study included patients in whom both PCT and LAC testing were conducted. This may have resulted in the selection of a population with greater severity of illness or who underwent more thorough testing. Thus, this population may not be representative of all patients presenting with acute cholangitis, limiting the generalizability of the findings. Lastly, PCT can be falsely elevated by comorbid conditions such as renal insufficiency or concomitant infection, potentially leading to confounding in results.

## Conclusions

The current study does not support the use of PCT to assess severity in AC with non-malignancy-related causes or prognosticate in-hospital outcomes. The findings suggest that PCT and LAC were both poor predictors of mortality, ICU admission, vasopressor use, and severe AC grade, and therefore should be interpreted with attention to the clinical context when used in this setting. Initial findings based on the current data, which require further verification, suggest that PCT may be useful in the evaluation of patients with malignant biliary strictures who present with AC. While the current recommendation by TG18 supports the limited use of PCT, large multicenter studies are necessary to definitively clarify its clinical applicability.
